# Establishing the Foundations of Emotional Intelligence in Care Companion Robots to Mitigate Agitation Among High-Risk Patients With Dementia: Protocol for an Empathetic Patient-Robot Interaction Study

**DOI:** 10.2196/55761

**Published:** 2024-10-04

**Authors:** Adeline Nyamathi, Nikil Dutt, Jung-Ah Lee, Amir M Rahmani, Mahkameh Rasouli, Donna Krogh, Erik Krogh, David Sultzer, Humayun Rashid, Hamza Liaqat, Riyam Jawad, Farhan Azhar, Ali Ahmad, Bilal Qamar, Taha Yasin Bhatti, Chet Khay, Jocelyn Ludlow, Lisa Gibbs, Julie Rousseau, Mahyar Abbasian, Yutong Song, Cheonkam Jeong, Sabine Brunswicker

**Affiliations:** 1 Sue & Bill Gross School of Nursing, University of California Irvine Irvine, CA United States; 2 Donald Bren School of Information and Computer Sciences, University of California Irvine Irvine, CA United States; 3 Smart Forward Rancho Palos Verdes, CA United States; 4 Department of Psychiatry and Human Behavior, and Institute for Memory Impairments and Neurological Disorders, University of California Irvine Irvine, CA United States; 5 NaviGAIT Irvine, CA United States; 6 Amore Senior Living Laguna Niguel, CA United States; 7 School of Medicine, University of California Irvine Irvine, CA United States; 8 Research Center for Digital Innovation, Purdue University West Lafayette, IN United States

**Keywords:** persons with dementia, empathy-based care companion robot, agitation, fall risk, artificial intelligence, AI

## Abstract

**Background:**

An estimated 6.7 million persons are living with dementia in the United States, a number expected to double by 2060. Persons experiencing moderate to severe dementia are 4 to 5 times more likely to fall than those without dementia, due to agitation and unsteady gait. Socially assistive robots fail to address the changing emotional states associated with agitation, and it is unclear how emotional states change, how they impact agitation and gait over time, and how social robots can best respond by showing empathy.

**Objective:**

This study aims to design and validate a foundational model of emotional intelligence for empathetic patient-robot interaction that mitigates agitation among those at the highest risk: persons experiencing moderate to severe dementia.

**Methods:**

A design science approach will be adopted to (1) collect and store granular, personal, and chronological data using Personicle (an open-source software platform developed to automatically collect data from phones and other devices), incorporating real-time visual, audio, and physiological sensing technologies in a simulation laboratory and at board and care facilities; (2) develop statistical models to understand and forecast the emotional state, agitation level, and gait pattern of persons experiencing moderate to severe dementia in real time using machine learning and artificial intelligence and Personicle; (3) design and test an empathy-focused conversation model, focused on storytelling; and (4) test and evaluate this model for a care companion robot (CCR) in the community.

**Results:**

The study was funded in October 2023. For aim 1, architecture development for Personicle data collection began with a search for existing open-source data in January 2024. A community advisory board was formed and met in December 2023 to provide feedback on the use of CCRs and provide personal stories. Full institutional review board approval was received in March 2024 to place cameras and CCRs at the sites. In March 2024, atomic marker development was begun. For aim 2, after a review of open-source data on patients with dementia, the development of an emotional classifier was begun. Data labeling was started in April 2024 and completed in June 2024 with ongoing validation. Moreover, the team established a baseline multimodal model trained and validated on healthy-person data sets, using transformer architecture in a semisupervised manner, and later retrained on the labeled data set of patients experiencing moderate to severe dementia. In April 2024, empathy alignment of large language models was initiated using prompt engineering and reinforcement learning.

**Conclusions:**

This innovative caregiving approach is designed to recognize the signs of agitation and, upon recognition, intervene with empathetic verbal communication. This proposal has the potential to have a significant impact on an emerging field of computational dementia science by reducing unnecessary agitation and falls of persons experiencing moderate to severe dementia, while reducing caregiver burden.

**International Registered Report Identifier (IRRID):**

PRR1-10.2196/55761

## Introduction

### The Impact of Dementia Among Older Adults

The prevalence of Alzheimer disease and related dementias (hereinafter “dementia”) has been increasing among older adults. In the United States, an estimated 6.7 million currently have dementia, and this number is expected to increase to 13.8 million by 2060 [1]. The majority of persons living with dementia experience neuropsychiatric symptoms of dementia [2] that are noncognitive, such as agitation, defined as inappropriate verbal, vocal, or motor activity [3]. Agitation is problematic because it can not only impact a person’s morbidity and mortality but also impair motor functions during events of agitation and increase the risk of falling and sustaining severe injuries and subsequent hospitalization [4,5]. Falls are a major cause of morbidity and mortality among older adults. Indeed, persons experiencing moderate to severe dementia are 4 to 5 times more likely to fall than older people without cognitive impairments [4,6,7]. Despite close observation and monitoring by caregivers, the onset of agitation is often unpredictable [8]. In fact, research has shown that agitation in persons with dementia in long-term care settings is associated with higher medication use and an increased likelihood of experiencing falls, fractures, infections, and other neuropsychiatric symptoms compared to older adults without agitation (odds ratio 1.58 for falls, 95% CI 1.41-1.77; *P*<.001); thus, it is important to develop an approach to effective management of agitation symptoms in this population to prevent falls [5].

### The Impact of Dementia on Caregivers

While much of the literature focuses on the fact that caring for persons experiencing moderate to severe dementia creates a heavy burden on caregivers, leading to caregiver burnout [9], only a few papers have highlighted ways in which caregivers of persons experiencing moderate to severe dementia can bring joy and happiness to their loved ones [10,11]. Furthermore, evidence has demonstrated that persons experiencing moderate to severe dementia can continue to express joy for minutes beyond remembering an event that was joyful [12]. However, more typically, the literature documents how informal caregivers (eg, spouses or adult children) for persons experiencing moderate to severe dementia experience chronic stress, depression, sleep disorders, poor health, low quality of life, and early mortality [13]. Family caregivers who live together with persons experiencing moderate to severe dementia show severe sleep disturbance compared to caregivers of persons with other health conditions, such as stroke or cancer [14]. Moreover, family caregivers of persons with dementia are twice as likely to experience depression as caregivers of persons without dementia [13,15]. Early mortality among spousal caregivers of persons with dementia is also not uncommon [16].

As a result, persons experiencing moderate to severe dementia are often removed from their homes due to worsening dementia and the heavy burden on caregivers [13,17]. Furthermore, despite evidence that person-centered intervention strategies may improve a person’s state [18], such strategies are not widely adopted, and the factors impacting the success of such interventions are not fully understood [9,19]. Anecdotes and personal experiences shared by caregivers as well as recent literature suggest that the success of person-centered care may depend upon a caregiver’s ability to relate to the patient and show empathy or the ability to imagine what they may be feeling or thinking [20]. However, the successful implementation of such care requires intense training, particularly when abusive behavior is present [9,19].

### The Current Status of the Use of Socially Assistive Robots as a Health Care Approach

Socially assistive robots are innovative solutions to deliver quality care for patients with dementia and to reduce agitation via verbal and nonverbal communication [21-23]. Social robots are typically equipped with onboard sensing technologies, including cameras and motion sensors, to sense the patient’s state and can support verbal and nonverbal communication with the person. Some robots come in the shape of an animal (ie, a dog) and have been shown to help persons with dementia build social relationships [22]. However, human-like robots (humanoids) that can demonstrate social patient-robot relationships can also have therapeutic effects, as measured in positive emotional responses [24]. Such social relationships are established through the presence of the social robot and sensorimotor interaction with the robot using haptics and vision (eg, touching and seeing the robot) and through the use of sound (eg, laughter). Currently, it is not clear whether and how robots might lower the risk of agitation through verbal communication. Furthermore, existing work on social robots fails to address 1 important issue: that agitation is associated with dynamically changing emotional disturbances [3].

Moreover, there is not sufficient research on how to design a care companion robot (CCR) that is equipped with such a conversational and empathetic intelligence that combines sound in the form of speech features with language-based interaction. Currently, there is a lack of understanding in how to best design emotionally aware robots that use empathetic conversations to trigger positive emotional change. Thus, research is needed that integrates both streams of research to close the gap on how emotional intelligence and empathetic conversations with caregiving robots can have therapeutic effects.

The specific research gaps we identified include three points: (1) The forms and the time-variant nature of emotions expressed by persons experiencing moderate to severe dementia are not sufficiently understood. (2) Existing technical solutions for emotion detection using data-driven machine learning (ML) algorithms, trained and fine-tuned on large amounts of data collected from healthy individuals and labeled, have not been sufficiently evaluated in the context of dementia, given the scarcity of emotional state data for patients experiencing moderate to severe dementia. While there is an active research community focused on detecting dementia based on language, speech signals, facial expression, and body movements, emotion detection for patients with dementia is not well studied. (3) Existing social robots lack the emotional intelligence to respond to person-specific emotional states through empathetic language-based communication, such as displaying understanding of a person’s feelings and emotions through words and speech features (eg, using a warm tone). Existing conversational robots use standard implementations of large language models (LLMs) without considering the challenges of fine-tuning such language models for greater empathy. Research on empathy alignment in general, and in the particular context of health care and dementia care, is scarce and requires significant exploration. The few available efforts to align LLMs either use simple prompt engineering or rely on data sets from social media platforms that have been labeled without a deep consideration of the language-specific properties of empathy [25-27]; (4) finally, beyond the technical research gaps related to ML and artificial intelligence (AI) and LLMs, the correlation relationship between different forms of emotions with motor impairments expressed in agitation and subsequent falls is unclear. While new research papers continue to be published on the impact of socially assistive robots on persons experiencing moderate to severe dementia [24], the research is still in a nascent stage. We believe that our study will provide significant contributions by building on recent research and addressing the aforementioned points. Table 1 articulates a lack of emotional intelligence and empathy-based conversations by existing robots versus our proposed CCR, which overcomes these limitations.

**Table 1:** Comparison of existing social robots’ limitations with the proposed care companion robot (CCR) improvements in emotional intelligence and empathy-based conversations.

Example	Limitations of existing social robot capabilities	Future capabilities of our social robot: the CCR
Paul, an 82-year-old resident with moderate to severe dementia, wakes up most mornings, forgetting how to use his walker as he gets out of bed.	He gets upset and angry trying to get out of bed. His social robot in the room, noticing a movement, approaches and starts talking using a standard dialogue flow with a normal voice pitch: “Hello Paul, how can I help you?” This makes Paul even more upset, worsening his emotional state. He becomes very angry and agitated, but the robot keeps asking: “How can I help you?” Paul’s agitation escalates. He shouts at the robot: “You can’t help me, stupid robot. I do not need your help.” Paul tries to get up without the walker and almost falls. Luckily, the caregiver observing Paul comes into the room and prevents the fall by calming him down using soft language and talks about exciting events in Paul’s past, about which he still has strong memories.	His CCR in the room senses a potential emotional shift ahead of time as Paul’s facial expression, body movement, and utterances indicate that he is becoming angry. When Paul starts trying to get out of bed, the CCR starts talking to Paul using soft language: “Good morning, Paul! Shall we go to breakfast?” At this point, the CCR pauses to ensure that the comment was understood and then continues the conversation: “Let’s use our walker this morning.” The CCR now shows an image of the walker which is close by, before speaking again: “But before we go, let us talk about your colleague at Boeing that you worked with.” Paul’s response: “You mean Alfred? He was such a great guy.” Paul continues his personal story, and the CCR responds, adding facts that Paul has forgotten. In the meantime, the caregiver, who had been informed about a potential onset of agitation, moves the walker closer to Paul so that he can get out of bed safely. Paul gets up. While walking to the breakfast room, the CCR and Paul are talking about Alfred. Paul has a happy start to his day.

### The Proposed CCR

A CCR is an advanced technological solution designed to provide assistance, support, and companionship for older adults, while addressing specific challenges associated with aging and cognitive decline. Equipped with autonomous indoor navigation, human-safe movement capabilities, and robust open robotics framework middleware (Open Source Robotics Foundation, Inc), the robot seamlessly navigates indoor environments while ensuring safety and reliability. Its multimodal interaction capabilities enable intuitive communication with users through speech, touch, and physical gestures using head and neck movement, facilitated by an Android tablet interface. With a suite of environment-monitoring sensors and customization options, the robot can be used to detect anomalies and adapt to various use cases, including the early detection of behavior changes indicative of conditions such as dementia. Powered by an onboard NVIDIA Jetson Orin device, it processes sensor data and executes tasks with high performance and efficiency, making it a valuable companion and caregiver support tool for older adults.

While the theoretical mechanism for storytelling in soothing agitation is yet to be articulated, Rios Rincon et al [28] reported that storytelling has helped to support memory, reminiscence, identity, and self-confidence—potential mechanisms under which storytelling may reduce agitation. Furthermore, other authors found that quality of life, depression, and the quality of the carer-patient relationship improved after the intervention with storytelling [29], perhaps because storytelling helps to build therapeutic relationships, bonds, rapport, comfort, and trust between older adults and caregivers [30], possibly leading to reduced agitation and fall risk. By providing mental stimulation, such as by storytelling, cognitive functions (eg, attention, executive function, and memory) may be improved and agitation, depression, and anxiety may be reduced [31,32].

### Objectives

This study describes the design and implementation of computational patient-robot interaction research designed to predict agitation and mitigate fall risk and tests the strategies in both a simulation laboratory with up to 50 undergraduate and graduate students and 12 persons experiencing moderate to severe dementia, who are residents of board and care facilities. The study aims to (1) collect and store new data sets used to train a CCR; (2) design new computational models focused on persons experiencing moderate to severe dementia; (3) develop new systems of empathy-based conversational models, using LLMs and theories of linguistics; and (4) pilot an empathy-focused intervention model for the CCR using a quasi-experimental intervention design and evaluate the conversational intervention longitudinally using mixed methods approaches.

## Methods

### Study Design

**Figure 1:**
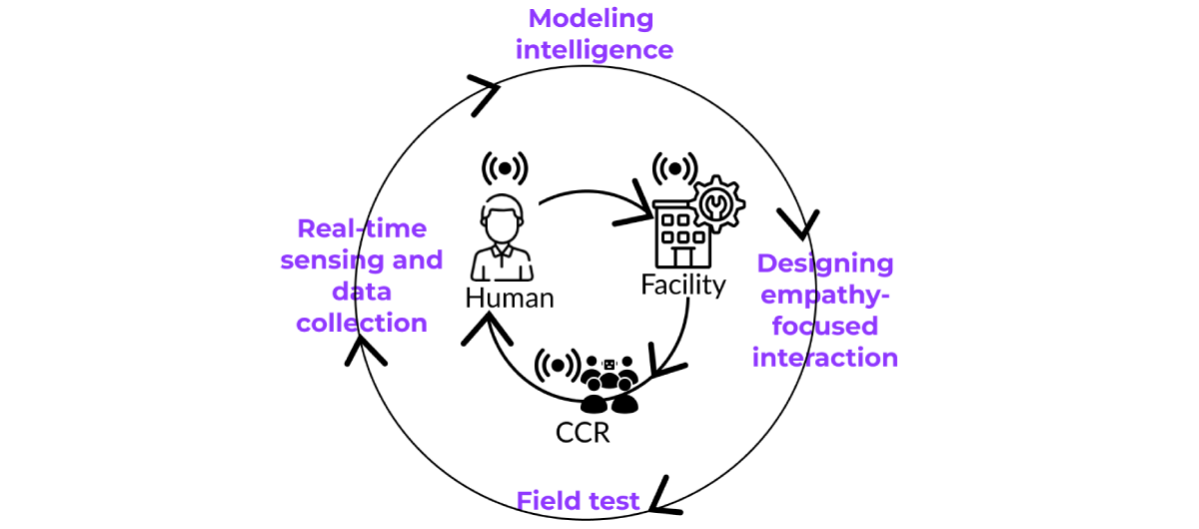
The design components of an empathy-focused conversation model to train a care companion robot (CCR) to assess the impact of conversational events on patients with moderate to severe dementia agitation.

The proposed 2-year study builds on the expertise of a multidisciplinary research team (ie, nursing, computer sciences, ML, AI, engineering, linguistics, and medicine). We will use computational ML and AI methods to first collect noninvasive data and develop models that can forecast a person’s emotional state, agitation level, and gait pattern in real time, using ML and AI and Personicle, a person-centric temporal activity or states database developed at the University of California Irvine (UCI) Institute for Future Health, and then test and evaluate the empathy-focused conversation model for the CCR in the community, using quasi-experimental and mixed methods approaches (Figure 1). We will use dynamic event analysis of conversations between the empathy-focused CCR and persons experiencing moderate to severe dementia to examine the impact of conversational events during a heightened emotional state on agitation level and gait pattern.

The aims of the study are as follows:

- Aim 1: collect and store granular Personicle data, using real-time visual, audio, and physiological sensing technologies in a simulation laboratory and at board and care facilities
- Aim 2: develop statistical models to understand and forecast the emotional state, agitation level, and gait pattern of a person experiencing moderate to severe dementia in real time using ML and AI and Personicle
- Aim 3: design and test an empathy-focused conversation model focused on storytelling
- Aim 4: test and evaluate the empathy-focused conversation model for the CCR in the community

Before the onset of the study, a community advisory board (CAB) will be formed to ensure that community feedback will be integrated into the logistics of the study, ensuring privacy and safety, as well as the relevance of the empathy-focused conversational models delivered by the CCR. The team will meet 3 times in the first year of the study. The CAB is designed to help guide CCR observation and modeling and, over the 2-year study, provide ongoing evaluative feedback on usability and acceptability collected from family and professional caregivers. All members of the CAB will be compensated for their time.

### Recruitment

Our study is being conducted at 4 board and care facilities in southern California, owned and operated by a certified family nurse practitioner. Each of the 4 facilities is licensed by the department of social services. Each board and care facility has an occupancy of 6 residents, who are cared for by a multidisciplinary team composed of nurse practitioners and medical assistants, with periodic visits by a physician and a music and exercise therapist. The residents are typically aged ≥65 years, and the majority typically experience moderate to severe Alzheimer disease; >50% also experience comorbidities, including hypertension and congestive heart failure, or have experienced a cerebrovascular accident. These conditions require 24-hour supervision and assistance due to safety concerns. The research staff work closely with the owner, who will ensure that all eligible residents (or their surrogates) receive a flyer about the study.

### Sample and Setting for Human Participant Engagement for Specific Aims 1, 3, and 4

For aim 1 (observation of persons experiencing moderate to severe dementia and interviews with family and professional caregivers), aim 3 (simulation laboratory role-plays with undergraduate and graduate students), and aim 4 (testing and evaluation of the impact of CCR modeling), relevant details are provided in the following subsections.

#### Aim 1: Persons Experiencing Moderate to Severe Dementia

The sample will include persons experiencing moderate to severe dementia residing at 4 board and care facilities in southern California, where a total of up to 24 patients reside, with turnover approximately every 6 months; approximately 25% (6/24) stay for 2 to 5 years. Current demographics reveal an equal distribution of male and female residents, who are predominantly White. The mean age is 88.3 (SD 5.72) years, and at least 1 family caregiver is involved. Of the 19 residents, 16% (3/19) have mild dementia, 58% (11/19) moderate dementia, and 37% (7/19) severe dementia. Particularly with residents who experience agitation, the risk of falls is a reality that is very concerning. We estimate, at a minimum, that 32% (6/19) residents will meet the eligibility criteria and remain at the facility throughout the 2-year study, with new patients admitted every month.

The inclusion criteria are as follows: (1) admitted by a physician as a person experiencing moderate to severe dementia, (2) residing at 1 of the 4 board and care facilities, (3) aged ≥40 years, and (4) consent provided by a legally authorized representative. The exclusion criterion is as follows: severe medical conditions that would limit participation in the study, including terminal illness.

#### Aim 1 and Aim 4: Family Caregivers

The sample will include up to 14 family caregivers of persons experiencing moderate to severe dementia who will be asked about their interest in participating in informal interviews to discuss any experiences in dealing with agitation and the fall risk of their family member and the joyful times their family member experienced (aim 1). For aim 4, up to 6 family caregivers whose family member with dementia is enrolled in the 3-month intervention and who provide consent will be administered 2 structured instruments at baseline and 3-month follow-up. They will also participate in a formal interview about how the CCR functioned in mitigating agitation and fall risk. The inclusion criteria are as follows: aged ≥18 years, English speaking, and self-reported family member of the person experiencing moderate to severe dementia.

#### Aim 1 and Aim 4: Professional Caregivers

The sample will include up to 5 professional caregivers of persons experiencing moderate to severe dementia who work at the board and care facility and who will be asked about their interest in participating in informal interviews to discuss any experiences in dealing with agitation and the fall risk of the person experiencing moderate to severe dementia they care for at the board and care facility. They will also be asked to share experiences of what makes the person experiencing moderate to severe dementia joyful versus sad (aim 1). For aim 4, up to 5 professional caregivers who care for the persons experiencing moderate to severe dementia enrolled in the 3-month intervention and provide consent will be administered 2 structured instruments at baseline and 3-month follow-up. They will also participate in a formal interview about how the CCR functioned in mitigating agitation and fall risk. The inclusion criteria are as follows aged ≥18 years, English speaking, and working at the board and care facility.

#### Aim 3: Undergraduate and Graduate Student Volunteers

For aim 3, approximately 50 student volunteers will be consented to spend approximately 1 hour in the simulation laboratory and will be trained by our investigative team to role-play a person with dementia who is experiencing emotions such as agitation. To capture authentic emotions, students may also be provoked to display emotions as well. The inclusion criteria are as follows: aged ≥18 years, enrolled undergraduate or graduate student at UCI, and English speaking.

### Ethical Considerations

#### Institutional Review Board Approval

Two institutional review board (IRB) requests have been submitted to the University of California Irvine (UCI) institutional review board. The first IRB request (non–human subject research), approved in December 2023 (UCI IRB #3795), related to accessing publicly available data sets containing multimodal and sensory input data for modeling emotion states, the degree of agitation and gait, as well as training the CCR for empathy-based conversational interventions for aims 2 and 3. The second IRB request (human subject research in the minimal-risk biomedical track) was approved in March 2024 for all aims (UCI IRB #4150). The IRB approval includes access to medical records and the observation of clinical care, and a partial waiver of Health Insurance Portability and Accountability Act (HIPAA) authorization has been requested.

#### Informed Consent

##### Family Caregivers and Health Care Professionals

At the beginning of the study, a study team member will provide family and professional caregivers with comprehensive information about the study and obtain their consent.

##### Participants With Dementia

As the participants with dementia might be cognitively impaired, the informed consent procedure will use a validated decision-making assessment tool [33] tailored to the cognitive abilities of participants with moderate to severe dementia. This tool will assess each participant’s capacity to comprehend the study objectives and procedure and determine their capability to provide informed consent regarding participation in the study. If the assessment indicates the participant’s lack of decision-making capacity to provide informed consent independently, a surrogate decision maker who is a legally authorized representative or a family member will be identified. The study team will inform the decision maker about the study, provide comprehensive details, and obtain their consent on behalf of the participant.

In addition, despite requiring surrogate consent, efforts will be made to obtain assent from the participant whenever possible. The assent process involves presenting information about the study in a manner that aligns with the participants’ cognitive abilities; seeking their agreement or disagreement (through words or nonverbal cues, including body language, facial expressions, gestures, or signs of agitation or discomfort when study-related discussions arise); and respecting their preferences to the best extent feasible.

However, because the study involves individuals with moderate to severe dementia, we anticipate fluctuations in decision-making capacity due to the natural progression of the disease. Consequently, participants’ ability to provide or withdraw informed consent may vary throughout the study duration. Therefore, because regular assessments of decision-making capacity are essential, qualified professionals will continue to evaluate participants’ ability to comprehend study information and make informed decisions. If, during the study, individuals demonstrate a diminished capacity to provide consent, the protocol outlines procedures for involving surrogate decision makers or legally authorized representatives to act in the participant’s best interests. It is essential to mention that participation in the study is completely voluntary, and all participants can opt out at any time without consequences.

#### Privacy and Confidentiality

The participants’ personal information and identifiers will be kept separate from the study data, and a code will be assigned to each participant. The deidentified data will be used for analysis and training purposes. After capturing audio and visual data of participants for whom they or their family members or legal guardians have provided consent, we will use the ARX data anonymization tool to anonymize sensitive personal data. All geographic locations, contexts, and environmental data will be abstracted to ensure that the collected data cannot be traced back to their original providers and will not be transmitted or stored in our cloud servers. In the cloud servers, the participants’ data will be processed using automated tools. We will make all relevant deidentified data and findings available after publication and will honor intellectual property rights.

The single electronic file containing participant identifiable information will be password protected and stored on a laboratory computer in the principal investigator’s locked laboratory, to which only the lead researcher, coresearchers, and study coordinators will have access. Research data will be stored electronically on a secure cloud-based data collection storage system maintained by the UCI Computer Science team led by a co-investigator.

As the audio recordings serve as primary training data for the CCR, it is crucial to preserve the integrity of identifiable audio recordings without transcription to preserve voice characteristics such as tone, intonation, and nuances in speech patterns, which are crucial for capturing the full complexity of human speech in developing ML algorithms. Therefore, the audio recordings will not be transcribed to prevent a loss or distortion of training data quality. However, deidentification procedures commence immediately after the audio recordings are obtained, ensuring minimal delay in protecting participant confidentiality.

The deidentification process for audio recordings involves several steps: (1) using specialized software such as Audacity or Adobe Audition designed for deidentification purposes; (2) using voice masking methods (including pitch shifting, time stretching, or applying filters to modify the frequency characteristics of the voice) within these tools to alter the pitch, tone, or timbre of the participants’ voices to make them less recognizable; (3) conducting quality checks to ensure that all identifiable information has been effectively removed or altered; (4) using speech intelligibility metrics such as signal-to-noise ratio, mean opinion score, and perceptual evaluation of speech quality to objectively measure the clarity of the modified audio; and (5) finally storing the deidentified audio recordings securely in a protected database to prevent unauthorized access. Similarly, preserving identifiable video recordings without transcription is crucial for comprehensive emotion and agitation detection as well as gait analysis, especially in training the CCR. These recordings contain critical contextual cues, facial expressions, body language, and environmental elements, and deidentification risks the integrity of developing precise training models. Therefore, the video recordings will not be deidentified.

#### Compensation

For aims 1 and 4, interviews will take place with family and professional caregivers, and a compensation of US $20 will be provided per interview. We anticipate conducting interviews with 8 to 14 family and professional caregivers for aim 1 and with 6 to 12 family and professional caregivers for aim 4. In addition, we will compensate approximately 50 students who will take part in the simulation laboratory role-plays (aim 3) with US $10 each.

### Research Description

#### Aim 1

For aim 1, we will collect and store granular Personicle data using real-time visual, audio, and physiological sensing technologies (including the CCR) to gain a deeper understanding of the emotional state, agitation level, and gait pattern of a person experiencing moderate to severe dementia over time.

##### Activity 1: Real-Time Data Collection and Sensing

The miles and outcomes involve creating (1) a database to store the hourly activities and behavioral states of a person experiencing moderate to severe dementia in real time and (2) a database to store their stories as episodic knowledge graphs.

###### Real-Time Sensing to Create Personicles

We will capture the behavioral states and patterns of a person experiencing moderate to severe dementia through real-time “lifelogging” using motion-sensing red-green-blue cameras with video capture and voice recording capacity (Microsoft Kinect), optical motion sensors (light detection and ranging [LIDAR] sensors), and remote photoplethysmography (rPPG) [34]. rPPG is a noninvasive optical technique used to estimate physiological parameters (eg, heart rate) remotely from a distance without the need for direct contact with the skin. The cameras and sensors will be installed in 2 bedrooms and the living room at 1 board and care facility at a time. We will begin observation of 2 patients at a time over at least a 2-month period, starting in month 2 and continuing until month 15, to capture the logs of 8 to 14 persons experiencing moderate to severe dementia. At least 6 to 8 persons experiencing moderate to severe dementia will remain for the full 2 years. In month 16, we will introduce the CCR and also use it as a real-time sensing device (using its onboard camera and LIDAR sensor). The CCR’s vision system is equipped with a human identification system. It can recognize >1 patient and track them (the CCR is also capable of identifying the caregivers). This allows the CCR to log the gait pattern, activities, and agitation level of the person under observation. This system will be configured during the start-up or onboarding process.

These sensing technologies will capture lower-level atomic, person-centric data—the “logs”—with a timescale of 1 minute that can be enriched with higher-level, more meaningful behavioral states using the models developed in activity 2. Specifically, we will model the emotional state, agitation level, and gait pattern of a person experiencing moderate to severe dementia and design a multimodel taxonomy and activity and states ontology, extending the existing Personicle, a person-centric temporal activity and states database developed at the UCI Institute for Future Health. For the purpose of recognizing agitation, our initial approach involves constructing models that leverage audiovisual sensing technologies. These models are designed to accurately recognize and categorize standard agitated behaviors as outlined in the Cohen-Mansfield Agitation Inventory [35,36]. This inventory includes a range of specific activities, such as kicking, grabbing, pushing, throwing objects, and screaming. After successfully capturing and classifying these activities, the next phase will focus on using these models to evaluate and quantify agitation levels in individuals with moderate to severe dementia. We will store activities or states at the minute level. This ontology will be used in activity 2 to find the relationships among the hourly or daily states and activities and to create a robust temporal activity model.

The ultimate goal of the CCR system is to develop a dynamic model that accurately captures an individual’s behavioral patterns. This model is intended to iteratively adapt and respond in a manner that positively influences and enhances these behaviors. In pursuit of this objective, a certain degree of tolerance for inaccuracy is considered acceptable, particularly in the precise identification of specific emotional states or the exact nature of agitated symptoms. This approach acknowledges that while exact details are beneficial, the primary focus is on the overall effectiveness of the model in improving behavioral outcomes.

###### Collecting Personal Stories

We will collect personal stories from our sample as input for activity 3. Our sensing devices will “lifelog” personal stories shared without probing when persons experiencing moderate to severe dementia converse with their caregivers at the board and care facilities. In addition, we will also elicit personal stories through informal interviews of the caregivers at least twice a week. By monitoring the behavioral state of persons experiencing moderate to severe dementia in their rooms, we can also categorize these events by emotion and agitation levels. Data will undergo manual review and labeling by our team of qualified clinical experts, performed offline for optimal focus. In addition, 2 to 3 interviews will be conducted with at least 1 family caregiver and the trained facility staff to collect information about their experiences about personal stories that have helped reduce agitation. We will also ask family members to share archival data (eg, videos of the persons experiencing moderate to severe dementia in the past as well as recordings of social and family events) that are representative of the past and positive or negative stories of the persons experiencing moderate to severe dementia. The unstructured natural language data will be translated into a knowledge graph to represent events or stories and additional attributes (eg, people and emotions) over time as an episodic relationship.

#### Aim 2

For aim 2, we will develop statistical models to understand and forecast the emotional state, agitation level, and gait pattern of a person experiencing moderate to severe dementia in real time using ML and AI.

##### Activity 2: Modeling Behavioral and Cognitive States

The miles and outcomes involve developing statistical models for modeling emotional states, agitation, and gait.

###### Modeling Emotional States

To model the emotional state of a person experiencing moderate to severe dementia, we will design multimodal ML and AI models using 4 types of data: natural language content, facial expression, body movements, and vocal features. We will focus on modeling emotions from each modality (eg, text as natural language content) and then integrate these models to create a comprehensive multimodal model. The goal is to evaluate the emotional content of a conversation at a particular point in time. In establishing these models, we consider both theoretical and technical aspects to ensure robustness. Specifically, in the case of the text modality, we will adapt existing theories (eg, the theory postulated by Parrott [37]) and ML and AI techniques (eg, pretrained models, such as Robustly Optimized Bidirectional Encoder Representations From Transformers Pretraining Approach [RoBERTa] [38], with fine-tuning) for inferring emotions from natural language to the context of dementia when modeling emotions using text extracted via speech-to-text techniques (for a comprehensive review, refer to the study by Zad et al [39]). This is especially pertinent to feature representation processes. In these processes, we will obtain word embeddings for emotions with the assumption that multiple emotional states can coexist simultaneously (for further information, refer to the study by MohammadiBaghmolaei and Ahmadi [40]). Our analysis will focus on 6 distinct emotional states: sadness, fear, joy, love, surprise, and anger. In the modeling process, we will pretrain our models on open-source data sets widely used for emotion recognition tasks in the natural language processing community (eg, the Interactive Emotional Dyadic Motion Capture Database [IEMOCAP] [41]) and then retrain them on data sets focused on conversation data from persons experiencing moderate to severe dementia, using both open-source data sets (eg, DementiaBank [42]) and our own (ie, the National Institute on Aging [43]).

Beyond the text modality, we will also train a deep learning model, using images and videos containing the facial expressions of a person experiencing moderate to severe dementia to infer emotions such as joy, sadness, or anger from their smiles, frowns, brow movements, and lip configuration. We will train our models using large image data sets such as Facial Expression Recognition 2013 [44], while also considering retraining them on data sets specific to patients with dementia [45,46]. In addition, videos and motion sensors will be used to infer emotions from a patient’s body movements. Audio data will be used to infer emotions based on markers such as speech rhythm, voice intensity, and other spectral features (eg, mel-frequency cepstral coefficients). Empathy modeling, including the alignment of LLMs using prompt engineering and reinforcement learning techniques, will also be conducted, considering the relationship between emotion and empathy.

Our research aims to make scientific contributions to ML and AI for emotional intelligence and empathetic conversation of CCRs in two main ways: (1) designing a new multimodal model for empathy classification that enables CCRs to sense a patient’s emotional state in real time; and (2) aligning LLMs for empathy using various techniques, such as prompt engineering, reinforcement learning, or a combination of both. For the first point, we plan to build a semisupervised ML model that uses a transformer architecture and pretrain it on healthy data sets. We will evaluate this baseline using dementia data sets carefully labeled by domain experts with respect to the emotional state of patients with dementia. We will then improve this baseline model by enhancing its self-supervision based on features identified from 4 modalities (ie, natural language content, facial expressions, body movements, and vocal features) in data collected from patients with dementia. We will examine different ML and AI models and evaluate their performance in both quantitative and qualitative ways.

Our major contribution will be the development of a semisupervised emotion classifier that integrates multiple data modalities, including text, speech features, facial images, and body movements. We will use established evaluation protocols and metrics, such as accuracy, precision, *F*_1_-score, recall, and confusion matrices, to assess and compare various models to evaluate this baseline model. We will complement these evaluation protocols with a qualitative evaluation and inspection of our emotion classifier. Furthermore, we will also examine the model’s run time, given the need for real-time evaluation of emotions.

For the second point, we will first evaluate existing open LLMs (eg, OpenLLaMA and NeuroLLaMa) using our own benchmarking data set and data sets published by others [27]. Building on this, we will then use prompt engineering methods to enhance the empathetic capabilities of the LLMs’ conversations. To assess the performance of these fine-tuned LLMs, we will use well-established metrics and protocols in alignment research, including *F*_1_-score, Bilingual Evaluation Understudy score, accuracy, precision, and recall. In addition to prompt engineering, we will leverage reinforcement learning to optimize the LLMs for empathy. Specifically, we plan to first use inverse reinforcement learning to develop a reward function for a highly empathetic caregiver.

###### Modeling the Degree of Agitation

We will develop an ML and AI model for the agitation exhibited by a person experiencing moderate to severe dementia that captures disturbances in verbal, vocal, or motor activity. Instead of relying on caregiver assessments, this model measures agitation in an objective way, using images, videos, and motion sensors, as well as collects nonintrusive physiological data using rPPG sensors (refer to the Real-Time Sensing to Create Personicles subsection). We first aim to extend recent novel research for modeling agitation via inertial body sensors to our setting of nonintrusive rPPG sensing [47]. We plan to enrich such models using image analysis following recent efforts to infer agitation via facial grimacing rather than body sensing [48]. We also aim to integrate vocal or verbal data to infer agitation states based on disturbances in verbal motor action. Using this multimodel ML and AI approach, we can enrich the Personicle of a person experiencing moderate to severe dementia by incorporating degrees and types of agitation (eg motoric functions). We will then build predictive models that allow us to predict both emotions and agitational states.

###### Modeling Gait

To facilitate continuous yet nonintrusive monitoring of gait and mobility, we will use a vision-based method to conduct comprehensive evaluations of crucial gait factors. Through the application of state-of-the-art computer vision techniques, the method will assess gait speed, step length, step width, knee flexion angle, and the Timed Up and Go test. The foundation of the vision-based method lies in its use of dependable and adaptable body pose estimation algorithms, which can be tailored to monitor and assess transitional activities. Thus, a more comprehensive comprehension of the gait patterns of the person experiencing moderate to severe dementia will be achieved, leading to a more precise identification of potential fall risks [49]. Integrating time-variant, person-level emotional, agitation, and mobility states will allow us to build models for understanding their causal relationships and prediction of high-risk events.

The system has been designed to operate in a normal home setting. Standard gait parameters such as gait speed, step length, step width, and cadence will be calculated using a combination of computer vision, statistical ML, and trigonometry. The Timed Up and Go test, which is a standard test, has been estimated from the aforementioned parameters using an ML model [50].

#### Aim 3

For aim 3, we will design an empathy-focused conversation model for successful human-robot intervention that considers the emotional state of a person experiencing moderate to severe dementia and associated events (using the outcomes of activity 1 and activity 2).

##### Activity 3: Designing Empathy-Focused CCR-Patient Interaction and Simulated Evaluation

The miles and outcomes involve developing natural language processing–based language models incorporating time-variant emotional states and events; an interface for empathy-based interaction (speech plus text display); and simulated validation of CCR usability as well as the impact of the conversational model on human emotional states, trust, and cognitive load.

In activity 3, we will focus on designing the empathy-focused intervention model for the CCR. The CCR is a social robot (standing 3 feet 9 inches tall and weighing 26 kg) that is primarily focused on acting as a companion for communication with the person experiencing moderate to severe dementia. In addition, it acts as a sensing device for activity 1. It is distinct from other social robots such as PARO, AIBO, or MINI, which are pets. The CCR is equipped with a 12-inch display and audio features to communicate with the person experiencing moderate to severe dementia via not only speech and sound (eg, music) but also text and visual content (eg, pictures and movies displayed). The software or hardware platform architecture of the CCR is modular and uses a widely adopted robot operating system. With state-of-the-art simultaneous localization and mapping (SLAM) and human-aware navigation capabilities, the CCR can expertly navigate complex environments while prioritizing the safety and well-being of humans.

Indeed, SLAM algorithms are used in many robotics applications in health care settings not only for research but also for day-to-day practice. Thus, the contribution to the field of SLAM research is not the priority of this research; instead, our focus lies on emotional intelligence and empathetic conversation. However, to ensure safety using SLAM, the team will advance existing SLAM models using vision for human recognition (in combination with other sensors such as those using LIDAR technology) to account for the dynamic environment in safety-critical conditions such as caregiving using visual SLAM, which integrates state-of-the-art research from fast-advancing computer vision research. Similar to the approach of Fang et al [51], we aim to improve the map construction of visual SLAM to allow for dynamic scene construction.

###### Design of an Emotionally Aware and Empathy-Based Conversational Model

To develop the CCR’s emotional intelligence, we will leverage the results of the activity described in the Modeling Emotional States subsection to design an AI model for understanding and predicting the emotional state and agitation level of a person experiencing moderate to severe dementia, using real-time sensing via the sensing technologies in the room. Using this AI model, the CCR will interact with the person experiencing moderate to severe dementia when the likelihood of an emotional disturbance or agitation increases, using an emotionally aware and empathy-focused conversation model. To realize such a conversational model, we will extend an open LLM in a way that enables the CCR to engage in empathetic storytelling with the person experiencing moderate to severe dementia. To do so, we will design a rule-based prompt engineering model that changes the degree of empathy of the CCR’s language. This empathy-prompting technique builds on recent research by Brunswicker et al [52] integrating AI with theories of empathy display in linguistics, reflecting the rules about how syntax and rhetoric in language display shape empathy in social conversations. In this research, the effect of AI-enabled empathy display in conversations with an artificial agent has been validated in randomized behavioral experiments with healthy participants exhibiting different degrees of emotional disturbances (eg, anger). We will advance these rule-based models in the context of patients with dementia to investigate the linguistic dimension of empathy for lowering the risk of agitation. Furthermore, we will integrate the LLM with an episodic knowledge graph that represents the personalized stories of the person experiencing moderate to severe dementia as connections of events, people, and activities [53]. This will enable the CCR to engage in personalized storytelling using empathetic language at the onset of an emotional arousal.

###### Design of the User Interface

Using the results of the activity described in the Design of an Emotionally Aware and Empathy-Based Conversational Model subsection, we will design a conversational interface that uses a dual-mode communication approach, incorporating both audio- and text-based interactions. This approach will allow the CCR to display empathy through its voice and on the display screen. Through these 2 ways, personal stories will be conveyed to the person experiencing moderate to severe dementia. Our design will focus on usability, aiming to reduce the cognitive load of the person experiencing moderate to severe dementia. To achieve this, we will draw upon established usability heuristics for interaction-enabled applications and revise our existing interface through an iterative and heuristic-based usability design process. This process will involve developing personas, which describe fictional or real individuals that represent end-user needs. These personas will support the product design of the audio and text interface.

In evaluating the usability of this interface, we will base our tests on established literature, specifically focusing on 2 widely used methods in the practitioner community: the 10 general principles for user interface design postulated by Nielsen [54,55] and the heuristics for conversational agent design proposed by Langevin et al [56]. We specifically plan to use the criteria proposed by Langevin et al [56] with some modification as the basis for the design of our audio and speech interface. Evaluation heuristics can be broadly categorized into 3 main areas: design (eg, aesthetic and minimalist design), content (eg, designing content for dialogue), and security (eg, data privacy). We will iteratively evaluate and update the interface to ensure optimal usability and performance accordingly.

###### Simulated Validation

Before evaluating our new empathy-focused CCR at the board and care facilities with persons experiencing moderate to severe dementia, we will work in the simulation laboratory of the UCI Sue & Bill Gross School of Nursing [57]. First, we will use the laboratory for simulated, randomized experiments to examine whether and how our newly designed conversational model for the CCR responds to videos and audio recordings of the board and care facility residents experiencing moderate to severe dementia collected in the activity described in the Activity 1: Real-Time Data Collection and Sensing subsection and played on a large screen to perform out-of-sample validation experiments related to the persons experiencing moderate to severe dementia. We will evaluate the CCR’s response time to approach the screen, its choice of a personal story, and its ability to align with the emotional state of the person experiencing moderate to severe dementia using both quantitative and qualitative techniques. Specifically, we will examine the content of the language, voice pitch, and the content logic of the stories. Afterward, we will perform exploratory evaluation studies as well as randomized controlled trials with healthy humans. We will carefully sample approximately 80 undergraduate and graduate students. Some will be provoked to be emotionally disturbed, using established scientific methods of emotion induction [58,59]. In the randomized controlled trials, we will use a simple 2×2 factorial treatment design (with CCR or without CCR and emotionally stable vs emotionally disturbed), with sessions lasting up to 1 hour. The goal is to evaluate usability, trustworthiness, helpfulness, cognitive load, and emotional states, as well as agitation levels before and after the interaction with the CCR (refer to the Activity 2: Modeling Behavioral and Cognitive States subsection) [58,59].

#### Aim 4

For aim 4, we will pilot an empathy-focused intervention model for the CCR using a quasi-experimental intervention design and evaluate the conversational intervention longitudinally using mixed methods approaches.

##### Activity 4: Pilot Implementation and Field Study

The miles and outcomes involve evaluating the impact of the CCR on the emotional state, agitation level, and gait pattern of 6 persons experiencing moderate to severe dementia and assessing the acceptability and usability of caregivers. We will use relational event analysis of empathy-focused conversations of patients and the CCR combined with pathway analysis of the persons experiencing moderate to severe dementia to examine dynamics over time.

###### Pilot Implementation

Starting in month 16, a total of 3 patients at a time will be interacting with 3 CCRs over a period of 3 months (1 introductory month for the patient and the CCR to become familiar with each other, while assessing for and mitigating agitation caused by the CCR as well as evaluating usability and acceptability periodically with family and professional staff every 2 weeks during a 2-month intervention period). Thereafter, an additional 3 persons experiencing moderate to severe dementia will be followed over the next 3 months, with observation ending in month 21 and final analysis and dissemination to follow. During the testing period, the CCR will be placed in the corner of the designated rooms; the family caregivers or staff will not need to program it in any way. At the onset of potential emotional disturbance, the CCR will move toward the person experiencing moderate to severe dementia, observe them closely, and start talking to them using empathy-focused conversations. The conversation will vary, depending on whether the person experiencing moderate to severe dementia expresses fear or anger. The CCR will use storytelling with the person experiencing moderate to severe dementia to prevent an onset of agitation when it detects relevant signals. If the CCR’s empathy-based conversations to prevent agitation are not successful, the traditional de-escalation strategy will be applied, such as facility caregiver support and behavioral intervention (caregivers are always notified of any agitation events), as well as, if necessary, the use of medication indicated and previously prescribed for the individual person experiencing moderate to severe dementia.

###### Evaluation (Quantitative)

We will measure the emotional state, agitation level, and gait pattern of the person experiencing moderate to severe dementia [48-53,57-65] using the models developed earlier (refer to the Activity 2: Modeling Behavioral and Cognitive States subsection). This computational and nonintrusive granular measurement is very novel for dementia research and allows us to associate conversational events with the emotional state of a person experiencing moderate to severe dementia. We will then perform a quantitative cross-sectional event-level analysis (comparing similar event periods within and without CCR interactions) and an intervention analysis using a dynamic event-state model for a few case settings, allowing us to understand how interactions with the CCR affect the outcomes of persons experiencing moderate to severe dementia over time (eg, relational event modeling). We will also use longitudinal analysis to assess perceived stress and health among family caregivers, with assessments at baseline (beginning at month 1 of the 3-month intervention with the CCR) and after the intervention (end of month 3), using the Caregiver Self-Assessment Questionnaire [66]. For the facility caregivers, we will use the Maslach Burnout Inventory [67] to assess emotional exhaustion and depersonalization.

###### Evaluation (Qualitative)

We will also conduct interviews with both types of caregivers to assess acceptability and usability, with questions to assess why the CCR did or did not impact the emotional state, agitation level, and fall risk. Thematic analysis on transcribed data from the caregivers will be conducted by qualitative investigators (AN and JAL), along with trained research staff, using a rigorous process [68]. In vivo coding will be generated from the first and second cycle codes of the participants’ responses [69]; these data will then be organized into a Microsoft Excel file.

The rigor of the iterative data analysis will be ensured by the trustworthiness of data (ie, credibility, confirmability, transferability, and dependability) [70]. Credibility will be ensured by delivering the sessions in safe and confidential areas to ensure open communication and audio recording and transcribing all conversations to ensure that the data are credible. Credibility will be further established by having 2 trained coders involved in the coding process, with a senior coder overseeing the entire process. Confirmability will be ensured through ongoing discussions of caring for the persons experiencing moderate to severe dementia with the caregivers and asking for feedback on previously collected data. Finally, dependability will be ensured by providing a detailed description of the methods used.

##### Data Management and Data Security

All study data will be entered and managed using REDCap (Research Electronic Data Capture; Vanderbilt University), a secure, web-based application designed to support research data entry and storage. Data will be entered using electronic tablets with secure socket layer protocol and protected by a firewall service provided by the Office of Information Technology. Data encryption and network-based access and key management services will be used for data storage.

## Results

### CAB Formation

To provide cultural nuances for the development and training of the CCR, a CAB was formed in December 2023. The CAB is composed of 6 members, 3 (50%) of whom are family caregivers of patients with dementia residing at the board and care facilities, with the other 3 (50%) being directors of, or professional caregivers working at, these facilities. During the first of several meetings that will occur several times over the period of the grant, the members expressed gratitude for the work that has been planned and highlighted the importance of the work in mitigating fall risk among patients with dementia.

### Activity 1: Real-Time Data Collection and Sensing

Architecture development for Personicle began in January 2024, using an existing open-source data set and foundations from the skeleton-based recognition module developed several years earlier [71]. To date, 16 actions (atomic, transition, and agitation markers) have been designed using client-server architecture. Accuracy to date is 99% on the test set and 96% on the validation set. Currently, we are developing a tracker with facial recognition capabilities for persons experiencing moderate to severe dementia and their caregivers.

Furthermore, to set the stage for capturing audio and visual images of the patients with dementia in their residential settings, camera stations were meticulously designed and customized according to the specific needs of the facilities we inspected. These internet-connected cameras are capable of detecting movement and start recording for 30 seconds when motion is sensed. In addition, a workstation computer was purchased in June 2024, and we will be using its graphics processing unit for training the models for aims 1 and 2; this workstation will also serve as substantial storage for all collected data. In addition, an automated and secure pipeline was developed, allowing the cameras to compress and transmit recorded videos, including point cloud images, and audio data to our high-performance workstation, where team members can securely access and analyze the data.

To test the functionality of our cameras, we conducted role-playing scenarios, simulating interactions between patients with dementia and caregivers, guided by dementia experts and facility observations. These simulations helped us identify potential issues, such as the high volume of the televisions in the rooms interfering with the detection of conversations between patients and caregivers, thereby limiting our ability to detect signs of agitation or emotional changes from audio cues. We addressed these issues by finding solutions to improve the accuracy of our recordings under such conditions. Through these activities, we created a simulated data set that mirrors real-life situations involving patients with dementia. This data set has been instrumental in training and testing our models to detect possible agitation or emotional changes, ensuring our system’s reliability and effectiveness in real-world applications.

### Activity 2: Modeling Behavioral and Cognitive States

First, as described in the Modeling Emotional States subsection, we initiated the development of ML and AI models by identifying open-source data sets and pretrained models relevant to our primary interests, such as emotion recognition and action recognition. These models serve as baselines for our project. This stage is particularly pertinent for problems related to classification or detection. Therefore, we focused on finding suitable open-source data sets and integrating them into our research objectives. The results of this stage are 2-fold: data set identification and construction and modeling.

Our initial objective was to establish an emotion classifier using various methods (eg, semisupervised method). To achieve this, we explored multiple data sets for emotion classification in the context of persons experiencing moderate to severe dementia and identified DementiaBank, which includes both text and audio files from 78 patients with dementia. As the data set was not labeled, 7 human labelers, 3 (43%) of whom are domain experts, were involved in the labeling process, during which we preprocessed the data set, including audio segmentation and utterance extraction from the text. During a training session, we built a guideline for labeling based on discussions with the labelers. Thereafter, the labelers labeled the data according to this guideline. The labeling activity has since been completed, and the labeled data are currently undergoing validation.

In parallel with the labeling activity, we established a baseline model for emotion classification using the IEMOCAP as well as the Multimodal EmotionLines Dataset (MELD) [72], open-source data sets widely used in emotion classification tasks. The IEMOCAP includes 151 dyadic dialogues, while the MELD consists of 1039 dyadic dialogues; both data sets contain text and audio data from healthy individuals. We obtained features from pretrained models, such as RoBERTa, for text and speech representation. Thereafter, multimodal modeling for emotion was implemented using transformers with self-attention and cross-attention layers, followed by emotion prediction. Through both quantitative and qualitative analyses of the results, we found that enhancing feature representations of each modality contributes to model performance.

Concurrently, a team from NaviGAIT and computer scientists from UCI are working on detecting agitation. We used the collected data and trained several models to detect different types of motions, such as sitting, standing, and so on, and annotate the video every 3 seconds. Currently, we are at the phase of training the model and improving its accuracy in detecting agitation. For audio, we are able to detect audio events using the Agitation Maker, which converts the audio into text and applies semantic scoring using a generative pretrained language model to obtain an agitation score. This work is under progress.

### Activity 3: Design of the User Interface

In terms of aim 3, the design use subgroup began developing personas by April 2024 and to date has developed 10 personas characterizing persons experiencing moderate to severe dementia that will be useful for training the models. The personas include the backgrounds of real persons residing at board and care facilities, with first names and pictures replaced, and includes brief descriptions of the persons, interests, personality, sleep quality, health conditions and medications prescribed, conversational ability, and entertainment and communication devices used, as well as what makes them happy or upset. Finally, more recently, we added behaviors to watch for that often leads to agitation and caregiver instructions for dealing with these behaviors. A second document was also developed that outlines the typical daily activities of each person experiencing moderate to severe dementia.

### Next Steps

Using client-server architecture, work is ongoing in predicting an action and activity every 3 seconds (refer to the Activity 1: Real-Time Data Collection and Sensing subsection). With multiple camera stations to be placed in position soon, the continued functionality of the cameras will be tested using role-playing. Furthermore, we will continue to advance the data set that mirrors real-life situations involving patients with dementia, which will be used to train and test our models to detect possible agitation or emotional changes, ensuring our system’s reliability and effectiveness in real-world applications. Ongoing design-use personas and workplans will be created to add rich premodel training data.

Once an appropriate baseline model is identified, we will proceed to gather data. In scenarios where suitable data sets are unavailable, we will undertake the collection of in-house data, tailored to our specific requirements. In instances where an open-source data set is available but inadequately labeled, our team will perform a meticulous process of relabeling the data to ensure alignment with our research needs. Following data acquisition and preparation, we will embark on the training and fine-tuning of our model. This process leverages the baseline model and is informed by the unique characteristics of our collected or curated data set.

The next step involves retraining the baseline model on the labeled data of persons experiencing moderate to severe dementia (ie, DementiaBank), after training and fine-tuning the baseline model with healthy data sets (ie, the IEMOCAP and the MELD), followed by both quantitative and qualitative analyses. This process includes building and testing various architectures, such as enhancing modality representations (eg, text representation), to improve model performance. Considering the nature of the data for emotion recognition (eg, imbalance and data size), we will adopt and develop various ML techniques (eg, attention weight) to mitigate potential issues that can arise. In parallel, we will establish a baseline model for empathy recognition using LLM alignments, including prompt engineering and reinforcement learning, considering the close relationship between emotion and empathy. Both quantitative and qualitative analyses of the results of all interim processes will be conducted. After scrutinizing these results and gaining insights from the analyses, we will improve our baseline models to build a robust empathic conversational model for the CCR, with data to be collected.

The subsequent phase involves rigorous testing of the trained model in various stages to ascertain its efficacy and reliability. It is imperative to note that the transition from a trained model to its application in real-world settings involves additional steps. Given our use of Jetson devices for edge computing, we will convert the trained model into TensorRT format. This conversion is a critical step to facilitate the deployment of the model on edge devices. Such deployment enables real-time inference while also adhering to privacy considerations, a crucial aspect of our research protocol.

Finally, aim 4 will be the culmination of our evaluation, wherein the CCRs will be stationed at the residences of the persons experiencing moderate to severe dementia enrolled in our study. Our goal is to then test and evaluate the empathy-focused conversation model for the CCR with the enrolled residents and gather qualitative data from both family and professional caregivers.

### Plan for Pretraining the Robot

We plan to develop a robust and efficient pretraining strategy for emotion detection in patients with dementia using publicly available data sets. We will first follow an unsupervised pretraining plan using speech feature extraction (eg, mel-frequency cepstral coefficients, pitch, and energy), visual feature extraction (eg, facial features related to emotion expressions, such as action units and facial landmarks), and physiological feature extraction (physiological signals such as heart rate and heart rate variability). The second step involves using supervised pretraining through fine-tuning the pretrained model on smaller, labeled data sets. Finally, we will further fine-tune the pretrained model on the final emotion detection task specific to patients with dementia.

Our review of current literature indicated the availability of publicly accessible data sets, including the Dem@Care, DementiaBank, and UCI Alzheimer’s Disease Research Center data set collections. The available data formats are text, voice, video, and sensor data (physiological signals). All data sets are in English, as the inclusion criteria were set and include healthy participants as well as participants experiencing dementia. The data sets were collected in controlled settings, such as research laboratories, or uncontrolled settings, such as home care or participants’ homes.

To enhance the existing literature review seamlessly, we will delve deeper into data sets that provide a nuanced perspective on dementia-related research; for example, 1 data set that stands out is Dem@Care, which serves as a resource with its collection of video and audio recordings captured in both laboratory and home settings using the Microsoft Kinect red-green-blue-D device. This data set goes further by including information on sleep patterns and physiological aspects. Another example is the Technology Integrated Health Management data set, which may be promising for remote health care monitoring because it leverages sensor data such as motion and sleep mat recordings. What makes this data set particularly interesting is its inclusion of labels for agitation events over a 6-hour period, allowing for correlations with sleep and activity data. Another valuable resource may be the Prompt database from i2 labs, which adds diversity by featuring video, audio, and facial expressions of participants in a home-based setting. This database provides a resource for training classification models.

By contrast, there is the DEAP data set, which focuses more on classification but does not specifically revolve around patients with dementia. For the dimension, we came across 2 data sets: Mobile Device Voice Recordings at King’s College London and the ADReSS Challenge. These data sets serve as voice banks for training audio-based models on individuals with dementia. However, both data sets require annotations to develop emotion-based audio models.

This careful choice of data sets, each with its own characteristics, not only expands the range of our exploration but also forms the basis for a thorough and detailed investigation into dementia across different methods and situations. As we progress, it will be important to consider the validity of such modeling and training using data sets from people without dementia or mild dementia and may not be fully applicable to persons with more advanced dementia. We will also consider cultural adaptation aspects in the CCRs, including customs, languages, and cultural social norms, in the designing phase. Engaging with communities to understand their values and preferences is key to ensuring that these robots are viewed as helpful companions rather than intrusive tools.

## Discussion

### Findings to Date

Worsening dementia places persons experiencing moderate to severe dementia at high risk for significant declines in cognition, emotion, and behavioral disturbances, including falls and serious injury due to unpredictable agitation. The findings of our study to date include for aim 1 and its activities, identifying, generating, and collecting public data sets for the training and testing of our models. We identified the key activities associated with persons experiencing moderate to severe dementia, including signs of agitation (such as standing up, pushing, and striking); we also located data sets for some of these activities and generated sample data sets with staff in our simulation laboratory and from the NaviGAIT team’s internal recordings to train AI models to detect key activities and extract relevant events. This activity resulted in a list of motion and audio events that we found to be correlated with agitation, along with their respective data sets. Finally, we acquired a computer workstation and camera designed for custom data set creation in preparing for aim 4 and testing real-world situations, along with capturing new data in our simulation laboratory.

For aim 2, we established a baseline model with benchmark data sets (ie, the IEMOCAP and the MELD) for the emotion classification task before incorporating the labeled data of persons experiencing moderate to severe dementia (ie, DementiaBank; the data were labeled by our team). We extracted features from each modality (ie, text and audio) using pretrained models (eg, RoBERTa) and leveraged them for multimodal modeling using transformer-based architectures. Furthermore, our analyses indicate that text representation is more challenging than speech representation. This may be due to the fact that emotion is not heavily dependent on text. Specifically, emotion is often implicit and not always conveyed through emotion-specific words such as *mad*. As a result, text-based emotion recognition can be more complex, especially because contextual information plays a crucial role in detecting emotions within text. This discrepancy in modality representation merits further discussion.

Given our findings and analyses of the results, our next steps include the following. First, we will navigate and develop embedding techniques (eg, ontology-based approaches) to enhance feature representations, especially text representation. In parallel, we will apply our baseline model to the labeled data and conduct quantitative and qualitative analyses of the results to gain insights into the language of emotion expression of persons experiencing moderate to severe dementia. Another step includes finding ways to mitigate potential problems caused by highly skewed data (eg, multiple “neutral” labels in the labeled data) because data quality is of paramount importance to model performance. Our interim goal is to improve text and speech modeling further. Following these steps, we will focus on empathy modeling and LLM alignments, as well as incorporating more modalities (eg, from video). We expect that these interim processes will significantly contribute to achieve our overarching goal: developing a robust empathic conversational model for the CCR, with data to be collected, ultimately benefiting persons experiencing moderate to severe dementia.

Finally, as part of aim 3, we have begun to develop personas and workplans focused on real persons experiencing moderate to severe dementia to help in pretraining the models, which will then set the stage for aim 4.

### Comparison With Prior Work

This proposed study is innovative in many ways. It uses a caregiving approach, developed with advice from community stakeholders, which will be tested both in our state-of-the-art simulation laboratory and in the community. The CCR will be designed to forecast and recognize signs of agitation and other emotional behaviors and, upon recognition, intervene with empathetic verbal communication. The CCRs will be assessed for their ability to objectively observe, record, analyze, and appropriately respond to persons experiencing moderate to severe dementia, relieving human caregivers of some of the time-consuming and stressful work they currently perform. Robot caregivers can potentially reduce the escalation of agitation among persons with moderate to severe dementia, because robots, unlike human caregivers, will not be prone to displays of distressed emotions that can exacerbate the distress and behavioral disturbance of persons experiencing moderate to severe dementia.

By the end of our study, we hope to develop a comparable CCR with our partnering board and care facilities where the persons experiencing moderate to severe dementia reside. Currently, we are developing the notification system that will enable the CCR to alert professional staff at the facilities when their support is needed for a patient.

The critical aspects we will be focusing on relate to whether we can train the CCR to predict agitation and be able to use storytelling to calm the individual while a support person is notified. We will be analyzing the communication and behaviors of individuals with dementia within their environments. This technique can then be applied to broader contexts, ensuring that the care strategies are versatile enough to benefit a wider range of individuals in various care settings (from homes to communities).

### Strengths and Limitations

The strengths of our study are significant. Our team has been collecting data from multiple sources, and the use of the Personicle data, gathered through real-time “lifelogging,” will be a novel approach. In terms of our emotion recognition model, by leveraging diverse benchmark data sets such as the IEMOCAP and the MELD, our model benefits from exposure to varied conversational contexts, enhancing its generalizability. Moreover, the multimodal approach, which extracts and integrates features from text and speech, allows for a comprehensive analysis of emotional information. Furthermore, using transformer-based architectures, our model effectively processes both local and global emotional context, handling the short-term and long-term dependencies inherent in conversations. In addition, our model’s performance benefits from the enhanced feature representation for each modality.

Despite these strengths, there are several prerequisites for the CCR to deliver on this vision. The first is the need for sufficient data to train and test the algorithmic models. There is a dearth of public data sets of persons experiencing moderate to severe dementia due in large part to the sensitive personal health information that would be revealed in an open format. Our team is overcoming these challenges by the combination of searching for and identifying new data sets; using the simulation laboratory to create data sets; and, for aim 4, testing the CCR at the board and care facilities with persons experiencing moderate to severe dementia.

Furthermore, building models for emotion classification comes with its own set of challenges. First, the data distribution of currently available benchmark data sets is highly skewed, with many “neutral” labels. Moreover, the diverse data sources of the benchmark data sets add to the complexity of the task. Specifically, the IEMOCAP includes dyadic conversations, while the MELD includes multiparty conversations. In addition, the IEMOCAP was created by actors in a laboratory setting, resulting in high-quality audio, whereas the MELD features conversations from the television show *Friends*. Consequently, model performance is sensitive to ambient factors, such as noise.

### Conclusions

Family caregivers of patients with dementia often experience high levels of stress when dealing with a loved one’s progressive dementia, often resulting in family disruption, with persons experiencing dementia being moved to community facilities. Moreover, even at these facilities, there is an inability of the professional staff to constantly observe and manage signs of agitation and fall risk. Our study has been making great progress in gathering existing data sets and collecting data via cameras and, subsequently, Internet of Things devices. These granular, personal, and chronological Personicle data will provide one of the first implementations of the framework envisioned by Jalali et al [73]. The development of novel models that receive input from persons experiencing moderate to severe dementia, algorithmically assess their emotional state and determine the appropriate intervention strategy, and deliver an empathetic conversational intervention is perhaps the most significant innovation in our proposal.

### Dissemination Plan and Future Directions

While this study will be conducted in a facility, agitation is an issue experienced by persons with dementia who reside in many types of facilities as well as at home. Thus, regardless of setting, the critical aspects we will be focusing on are whether we can train the CCR to predict agitation and be able to use storytelling to calm the individual while a support person is notified. We will be analyzing the communication and behaviors of individuals with dementia within their environments. This technique can then be applied to broader contexts, ensuring that the care strategies are versatile enough to benefit a wider range of individuals in various care settings (from homes to communities).

Future research will focus on developing the baseline model by fine-tuning and enhancing feature representations. This includes assessing the model both quantitatively and qualitatively. Thereafter, we will retrain the model with the data from DementiaBank, which has been carefully labeled by our domain experts. We will explore and develop various model architectures (eg, semisupervised models) to analyze emotions in both healthy and dementia data sets and evaluate the model performance, after which features from another modality, video, will be incorporated into the model. This proposal thus has the potential to have a significant impact on an emerging field of computational dementia science and on society by reducing episodes of falls, injuries, and unnecessary hospitalizations of persons experiencing moderate to severe dementia, while helping to relieve caregiver burden.

## Abbreviations

AI: artificial intelligence
CAB: community advisory board
CCR: care companion robot
HIPAA: Health Insurance Portability and Accountability Act
IEMOCAP: Interactive Emotional Dyadic Motion Capture Database
LIDAR: light detection and ranging
LLM: large language model
MELD: Multimodal EmotionLines Dataset
ML: machine learning
REDCap: Research Electronic Data Capture
RoBERTa: Robustly Optimized Bidirectional Encoder Representations From Transformers Pretraining Approach
rPPG: remote photoplethysmography
SLAM: simultaneous localization and mapping
UCI: University of California Irvine

## Data Availability

Data sharing is not applicable to this study because no data sets have been generated or analyzed yet for the preparation of this protocol manuscript.
